# The Incidence, Outcomes, and Risk Factors of Secondary Poor Graft Function in Haploidentical Hematopoietic Stem Cell Transplantation for Acquired Aplastic Anemia

**DOI:** 10.3389/fimmu.2022.896034

**Published:** 2022-05-09

**Authors:** Fan Lin, Tingting Han, Yuanyuan Zhang, Yifei Cheng, Zhengli Xu, Xiaodong Mo, Fengrong Wang, Chenhua Yan, Yuqian Sun, Jingzhi Wang, Feifei Tang, Wei Han, Yuhong Chen, Yu Wang, Xiaohui Zhang, Kaiyan Liu, Xiaojun Huang, Lanping Xu

**Affiliations:** ^1^ Beijing Key Laboratory of Hematopoietic Stem Cell Transplantation, National Clinical Research Center for Hematologic Disease, Collaborative Innovation Center of Hematology, Peking University Institute of Hematology, Peking University People’s Hospital, Beijing, China; ^2^ Peking-Tsinghua Centre for Life Sciences, Beijing, China

**Keywords:** secondary poor graft function, acquired aplastic anemia, haploidentical hematopoietic stem cell transplantation, risk factors, cytomegalovirus (CMV), graft-versus- host disease

## Abstract

Secondary poor graft function (sPGF) increases the risk of life-threatening complications after hematopoietic stem cell transplantation (HSCT). The incidence, clinical outcomes, and risk factors of sPGF have not been elucidated in haploidentical (haplo-) HSCT for acquired aplastic anemia (AA) patients. We retrospectively reviewed 423 consecutive AA patients who underwent haplo-HSCT between January 2006 and December 2020 and report a 3-year cumulative incidence of 4.62% (95% confidence interval [CI]: 3.92%-10.23%) of sPGF. While no primary PGF occurred. The median time to sPGF was 121 days (range 30-626 days) after transplantation. To clarify the risk factors for sPGF, 17 sPGF cases and 382 without PGF were further analyzed. Compared to patients without PGF, the 2-year overall survival was significantly poorer for sPGF patients (67.7% vs 90.8%, p =.002). Twelve sPGF patients were alive until the last follow-up, and 7 achieved transfusion independency. The multivariable analyses revealed that later neutrophil engraftment (OR 2.819, p=.049) and a history of refractory cytomegalovirus viremia (OR=7.038, p=.002) post-transplantation were associated with sPGF. There was weak evidence that a history of grade 3-4 acute graft-versus-host disease increased the risk of sPGF (p=.063). We advocated better post-transplantation strategies to balance the risk of immunosuppression and viral reactivation for haplo-HSCT in AA patients.

## 1. Introduction

Survival after hematopoietic stem cell transplantation (HSCT) in acquired aplastic anemia (AA) has been remarkedly improved over the last three decades. Haploidentical (haplo-) HSCT provides easily available donors for patients with AA and guarantees a favorable engraftment rate (1–3). Stable hematopoietic recovery is the key point of successful HSCT for AA. However, hematologists have noticed that even if the patients achieved initial hematopoietic reconstitution and maintain complete donor-originated hematopoietic cells, they may develop intractable multilineage cytopenia afterwards (4, 5). This is defined as secondary poor graft function (sPGF), and it occurs in 5-27% of post-transplantation cases (6). Patients with sPGF lose their initial hematopoietic reconstitution, which leads to increased risks of severe infection, major bleeding events, and other life-threatening complications after transplantation. The therapeutic options for sPGF are limited, and the prognosis remains poor.

Studies have reported several risk factors for sPGF. Cytomegalovirus (CMV) reactivation and graft-versus-host disease (GvHD) are the two most recognized risk factors (6). Other potential risk factors include haplo-HSCT setting, recipient age, conditioning regimen, Epstein-Barr virus (EBV) infection, etc. (7–9) However, these conclusions were limited by heterogeneity in disease categories and transplantation settings. The clinical outcomes and risk factors of sPGF have not been elucidated in haplo-HSCT for AA patients. Based on the largest-scale AA cases receiving haplo-HSCT, we herein retrospectively analyzed the incidence, outcomes, and risk factors of sPGF.

## 2. Method

### 2.1 Study Population

In this study, we reviewed 423 consecutive AA patients who underwent haplo-HSCT as first HSCT between January 2006 and December 2020 at Peking University People’s Hospital (PKUPH). Written informed consent was obtained from each patient before transplantation. The study protocol followed the Declaration of Helsinki and was approved by the Ethics Review Committee of PKUPH. The cumulative incidence of PGF was estimated based on the whole cohort. Then, patients who developed graft failure after haplo-HSCT (primary graft failure n=2, and secondary graft failure n=3; 1.18% in total) or died of any cause within 28 days post-transplantation (n=19, 4.49%) were excluded from further analysis.

### 2.2 Transplantation Protocol

All patients received mixed graft infusion of granulocyte colony-stimulating factor (G-CSF) mobilized bone marrow (BM) and peripheral blood (PB) stem cells except for three cases (0.67%) in which only PB grafts were infused. The conditioning regimen for acquired AA patients included: (1) BuCy-ATG conditioning including busulfan (Bu, 3.2 mg/kg daily on days -8 and -7), cyclophosphamide (Cy, 50 mg/kg daily on days -5 to -2), and rabbit antithymocyte globulin (rATG, 2.5 mg/kg daily on days -5 to -2, from SangStat, France); and (2) the BuCy^low^Flu-ATG regimen consisting of Bu (0.8 mg/kg 4 times daily on days -8 and -7), Cy (25 mg/kg daily on days -5 to -2), Flu (30 mg/m2 daily on days -6 to -2), and rATG (2.5 mg/kg daily on days -5 to -2) (10, 11). The prophylaxis of GvHD was described elsewhere (10).

All patients received ganciclovir (GCV)-based preemptive therapy when CMV viremia was diagnosed. Foscarnet and immunoglobulin were administered if patients were intolerant to GCV or had an increase in CMV DNA copy after receiving full dose of GCV for 1 week. Refractory infections were treated with CMV-specific T cells at the discretion of physician. Once EBV viremia developed, a reduction in the dose of immunosuppressants would be taken for patients without or less than grade II aGvHD. Rituximab was applied to progressive EBV infection based on physician’s decision and EBV-associated post-transplant lymphoproliferative disease. For refractory CMV and EBV co-reactivation, CMV/EBV-specific T cells would be prepared and infused (12, 13).

### 2.3 Protocol for DSA Detection and Desensitization

The anti-human leukocyte antigen (HLA) antibody was routinely examined pre-transplantation. Detection of donor-specific antibody (DSA) was performed according to an established protocol. DSA-positive patients (2000 ≤ mean fluorescence intensity [MFI] < 10000) were given rituximab 3 days before graft infusion. If available, DSA-negative umbilical cord blood was also infused prior to infusion of allogeneic grafts (14, 15). No patients in this study had a DSA MFI of ≥ 10000.

### 2.4 Evaluation and Definitions

Poor graft function (PGF) was defined as sustained cytopenia of 2 or 3 lineages (neutrophil count <.5 × 10^9^/L, hemoglobin < 70 g/L, and platelet count < 20 × 10^9^/L) for over 2 weeks with full donor chimerism of > 95%, hypoplastic-aplastic BM, and absence of severe GvHD, active infection and drug toxicity. Primary PGF referred to PGF that failed to achieve initial engraftment, and sPGF was defined as a decrease of blood counts after prompt recovery (16, 17). Chimerism analysis was evaluated using PB at 1, 2, 3, 6, 12 months post-transplantation and at annual outpatient visits thereafter. The analysis of chimerism was also performed every time when the blood counts obviously fluctuated. Immune reconstitution within 30, 60, and 90 days after transplantation, including CD3+, CD4+, and CD19+ cells, was documented.

Neutrophil engraftment was defined as the first of 3 consecutive days when the absolute neutrophil count reached the level of >.5 × 10^9^/L without G-CSF stimulation. Platelet engraftment was defined as the first of 7 consecutive days when the platelet count was > 20 × 10^9^/L, independent of platelet infusion. Both acute GvHD (aGvHD) and chronic GvHD (cGvHD) were diagnosed and graded based on published criteria (18, 19). CMV and EBV DNAemia ≥ 1 × 10^3^ genome copies/mL were considered positive using real-time quantitative PCR (12, 13). Refractory CMV reactivation was defined as growing CMV-DNA copies or viral load of the same level after at least 2 weeks of appropriately dosed antiviral therapy. Recurrent CMV reactivation was diagnosed when a patient who had previous evidence of CMV viremia and had no virus detected for at least 4 weeks during active surveillance developed a new CMV viremia (20, 21). Overall survival (OS) was defined as the time from the date of haplo-HSCT to death or the last follow-up.

### 2.5 Statistical Analysis

The last follow-up for all survivors was April 1^st^, 2021. All clinical data were analyzed using R software (version 3.6.3, https://www.r-project.org) and Prism 8 (GraphPad Software, La Jolla, CA). The continuous variables were summarized as median (range) for nonnormally distributed data and compared using the Mann-Whitney test, and the categorical variables were expressed as count and percentage and compared using the chi-square test or Fisher’s exact test. The cumulative incidence rate (CIR) of sPGF or engraftment was estimated with death as a competing event and performed using the “cmprsk” package. Virus reactivation and aGvHD that developed before sPGF were calculated. The CIR of GvHD and virus reactivation were also estimated competing with events including death and PGF. In addition, the Kaplan-Meier method was used to estimate survival curves. Univariable analysis was performed based on logistic regression models, and potential risk factors (p <.10) were further analyzed in multivariable analysis. The infused doses of CD34+ cells (stratified by median) and conditioning regimen (Flu-based vs noFlu) were included with interest in multivariable analysis regardless of their p-values. Before multivariable analysis, we examined the correlation and multicollinearity among potential risk factors. A multivariable logistic regression was used to determine the independent effect of the included factors. All statistical tests were 2-sided, and a p-value <.05 was considered statistically significant.

## 3. Result

### 3.1 Incidence and Characteristics of sPGF

In the whole cohort, no primary PGF and mixed chimerism were observed in our study. The 3-year CIR of sPGF was 4.62% (95% CI: 3.92%-10.23%). All patients with sPGF had neutropenia of <.5 × 10^9^/L and thrombocytopenia of < 20 × 10^9^/L with or without red blood cell transfusion dependence.

The characteristics of sPGF (n=17) and noPGF (n=382) patients are summarized in **Table 1**. The median time of sPGF was 120 (range 30-626) days post-transplantation. Compared to patients without PGF, patients with sPGF had marginally longer interval from disease onset to haplo-HSCT (p=.057). Except for the above parameters, patients with sPGF and those without PGF had equivalent recipient, donor, and graft characteristics.

**Table 1:** Characteristics of sPGF and noPGF groups and post-transplantation outcomes.

	sPGF (n=17)	noPGF (n=382)	p-value
Recipient age, years, median (range)	26 (2-55)	14 (1-54)	.261
≥16, n (%)	10 (58.8)	187 (49.0)	.298
Male, n (%)	10 (58.8)	220 (57.6)	.920
vSAA, n (%)	1 (5.9)	88 (23.0)	.136
PNH clone, n (%)	2 (11.8)	36 (9.4)	.669
AA course, month, median (range)	12 (3.0-192.0)	15 (1.0-468.0)	.301
SAA course, month, median (range)	11.5 (3.0-108.0)	8 (0.5-264.0)	.057
Prior CSA plus ATG, n (%)	1 (5.9)	73 (19.1)	.217
Transfused RBC, unit, median (range)	30 (8-140)	20 (0-600)	.202
Transfused PLT, unit, median (range)	24 (2-81)	15 (0-248)	.188
SF level, ng/mL, median (range)	2586.0 (388.0-8804.0)	1719.0 (8.8-20251.0)	.424
Donor age, years, median (range)	40 (18-61)	38 (8-65)	.217
Donor source, n (%) parent sibling offspring collateral	14 (82.4) 2 (11.7) 1 (5.9) 0 (0.0)	297 (77.7) 63 (16.5) 20 (5.2) 2 (0.6)	.998
Donor-recipient sex match, n (%) male to male male to female female to male female to male	7 (41.2) 6 (35.3) 1 (5.9) 3 (17.6)	167 (43.7) 124 (32.5) 35 (9.2) 56 (14.7)	.961
HLA match, n (%)^*^ 1/6 2/6 3/6	1 (5.9) 3 (17.6) 13 (76.5)	14 (3.7) 63 (16.5) 305 (76.5)	.562
Anti HLA-I, n (%) positive negative missing data	2 (11.7) 13 (76.6) 2 (11.7)	62 (16.2) 249 (65.2) 71 (18.6)	.756
Anti HLA-II, n (%) positive negative missing data	2 (11.7) 13 (76.6) 2 (11.7)	28 (7.3) 283 (74.1) 71 (18.6)	.620
DSA, n (%) MFI < 2000 MFI ≥ 2000 negative missing data	1 (5.9) 1 (5.9) 13 (76.5) 2 (11.8)	17 (4.5) 10 (2.6) 281 (73.6) 74 (19.4)	.478
ABO match, n (%) match minor mismatch major mismatch bidirectional mismatch	12 (70.6) 1 (5.9) 3 (17.6) 1 (5.9)	176 (46.1) 77 (20.2) 81 (21.2) 48 (12.6)	.283
Conditioning regimen, n (%) Flu-based noFlu	6 (35.3) 11 (64.7)	81 (21.2) 301 (78.8)	.225
Graft characteristics			
MNCs (×10^8^/kg), median (range)	9.49 (7.02-17.35)	9.63 (5.07-44.52)	.696
CD34+ cells (×10^6^/kg), median (range)	2.96 (0.96-15.05)	2.87 (0.14-22.47)	.671
CD4+/CD8+ in BM, median (range)	1.31 (0.41-2.34)	1.17 (0.09-14.80)	.998
Neutrophil engraftment, n (%)	17 (100)	381 (99.7)	1.000
28-day engraftment rate (%)	94.1	99.7	.081
time of engraftment, day, median (range)	15 (11-31)	12 (9-22)	**.005**
Platelet engraftment, n (%)	11 (64.7)	368 (96.3)	**<.001**
100-day engraftment rate (%)	52.9	94.2	**<.001**
time of engraftment, day, median (range)	22 (10-338)	15 (5-180)	.167
History of early CMV reactivation, n (%)			
within 28 days	2 (11.8)	107 (28.0)	.158
within 100 days	13 (76.5)	276 (72.3)	.701
History of refractory CMViremia, n (%)	4 (23.5)	16 (4.2)	**.007**
History of recurrent CMViremia, n (%)	2 (11.8)	44 (11.5)	.752
History of early EBV reactivation, n (%)			
within 28 days	1 (5.9)	11 (2.9)	.467
within 100 days	2 (12.2)	55 (14.4)	.921
History of aGvHD, n (%)			
grade 2-4 aGvHD	5 (29.4)	120 (31.5)	.778
grade 3-4 aGvHD	4 (23.5)	33 (8.6)	**.040**
Follow-up time for survivor, days, median (range)	758 (94-1767)	1012 (94-4874)	

sPGF secondary poor graft function, AA aplastic anemia, SAA severe aplastic anemia, vSAA very severe aplastic anemia, PNH paroxysmal nocturnal hemoglobinuria, CSA cyclosporine A, ATG antithymocyte globulin, RBC red blood cell, PLT platelet, SF serum ferritin, HLA human leukocyte antigen, DSA donor-specific antibody, MFI mean fluorescent intensity, Flu fludarabine, MNCs mononuclear cells, BM bone marrow, PPR poor platelet reconstitution, CMV cytomegalovirus, EBV Epstein-Barr virus, aGvHD acute graft-versus-host disease. P value <.05 was emphasized with bold fonts.

^*^ HLA-A, -B, and -DR matching was included.

### 3.2 Transplantation Outcomes and Complications Before sPGF

**Figure 1:**
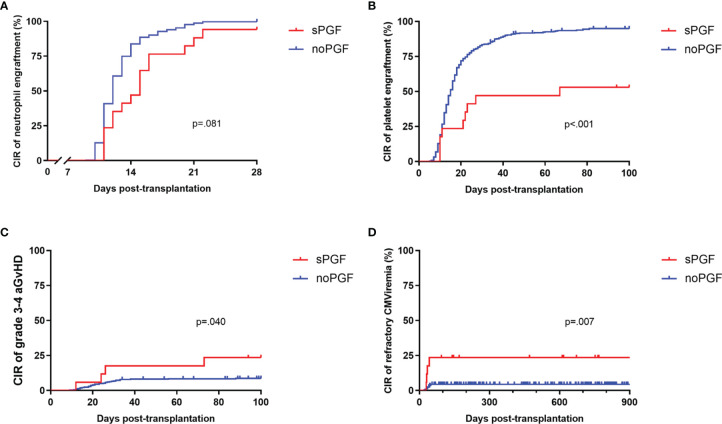
The CIR of transplantation outcomes and complications in sPGF patients and noPGF patients: **(A)** 28-day neutrophil engraftment, **(B)** 100-day platelet engraftment, **(C)** grade 3-4 aGvHD, **(D)** refractory CMV viremia. Patients were censored when they were diagnosed with sPGF.

Except for one patient who died of thrombotic microangiopathy at day +34, all patients included in this study were confirmed to achieve neutrophil engraftment. The CIR of 28-day neutrophil engraftment for sPGF patients was marginally lower than that for patients without PGF (94.12% [95% CI: 88.82%-96.95%] vs 99.74% [95% CI: 99.64%-99.81%], p=.081, **Figure 1A**). The median time of neutrophil engraftment was delayed in the sPGF group (15 [range 11-31] vs 12 [range 9-22] days, p=.005, **Table 1**). Additionally, the CIR of 100-day platelet engraftment was lower in patients who subsequently developed sPGF (52.94% [95% CI: 28.89%-72.19%] vs 94.92% [95% CI: 94.11%-95.62%], p<.001, **Figure 1B**).

Prior grade 3-4 aGvHD was also more likely to be observed among sPGF patients (p=.040, **Figure 1C**). The incidences of early CMV and EBV reactivation, either within 28 days (p=.158 and.467, respectively) or within 100 days (p=.701 and.921, respectively), were similar between groups. More patients with sPGF had a history of refractory CMV viremia (p=.007, **Figure 1D**).

**Figure 2:**
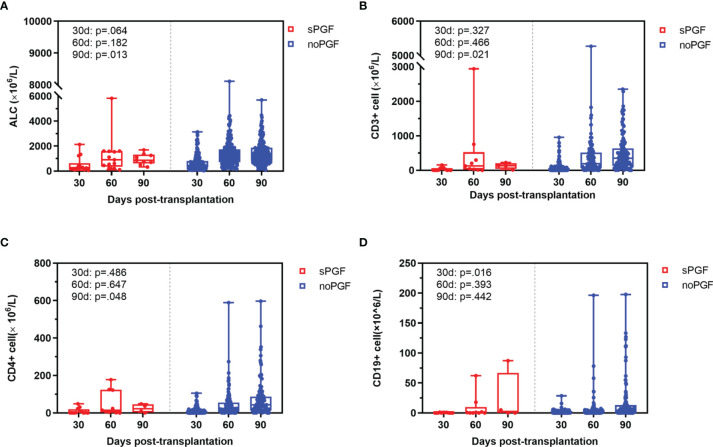
Immune reconstitution at day 30, 60, and 90 in sPGF patients and noPGF patients: **(A)** absolute lymphocyte count (ALC), **(B)** CD3+ T cell, **(C)** CD3+CD4+ T cell, **(D)** CD19+ B cell.

T cell reconstitution before sPGF was comparable at day 30 and day 60. However, CD19+ B cell reconstitution was delayed in patients who developed sPGF later (p=.016, at day 30). At day 90, patients in the sPGF group had a trend toward poorer T cell reconstitution, although the data available was limited. In addition, lower levels of lymphocytes were observed at day 90. (**Figure 2**)

### 3.3 Treatment and Outcomes of sPGF

Compared to the noPGF group, the sPGF group experienced significantly poorer 2-year OS (67.71% [95% CI: 38.83%-85.15%] vs 91.46% [95% CI: 88.07%-93.93%], p =.002). Twelve sPGF patients were alive until the last follow-up, and 7 of them were transfusion independent. Infection was the leading cause of death (4/5) in sPGF group, and one died of intracranial hemorrhage.

All patients with sPGF received supportive treatment, G-CSF, blood transfusion, androgens, immunosuppression agents, etc. Only 1 patient had spontaneous hematopoietic recovery without further treatment. Among the other 4 patients who received only supportive treatment, 3 died of infection. Two patients additionally received eltrombopag. One of them became transfusion-independent and the other, although remained transfusion-dependent, had extended the transfusion interval.

**Figure 3:**
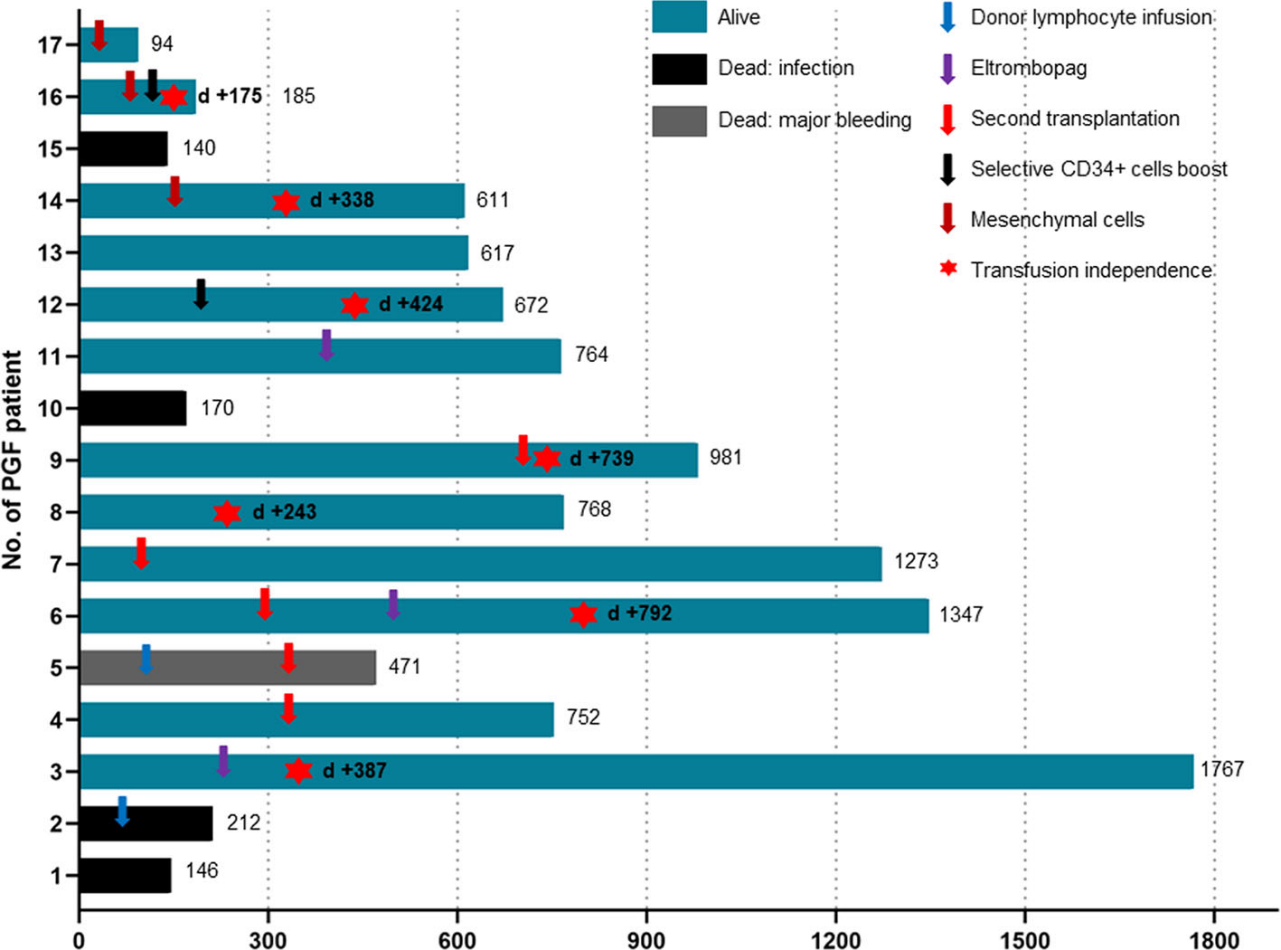
Treatment and outcomes in sPGF patients.

Ten sPGF patients received salvage treatments in forms of cellular therapies (**Figure 3**). Two were infused with donor lymphocytes with no improvement in hematopoietic function. One of them died of infection, and the other died of intracranial hemorrhage following a second transplantation from the original donor. Upfront second transplantation was applied in another 4 cases. Three received grafts from their original donors and experienced prolonged isolated thrombocytopenia, one of whom discontinued blood transfusions after treatment with eltrombopag. The other one received second transplantation from another haploidentical donor (mother) and achieved sustained transfusion independence. Of the three sPGF patients who received mesenchymal cell infusions, only 1 patient achieved transfusion independence and the other 2 patients had no response. Notably, selective CD34+ cell boost successfully resulted in normal blood counts in 2 patients with sPGF.

### 3.4 Risk Factors for sPGF

#### 3.4.1 Pre- or Post-Transplantation Variables

**Figure 4:**
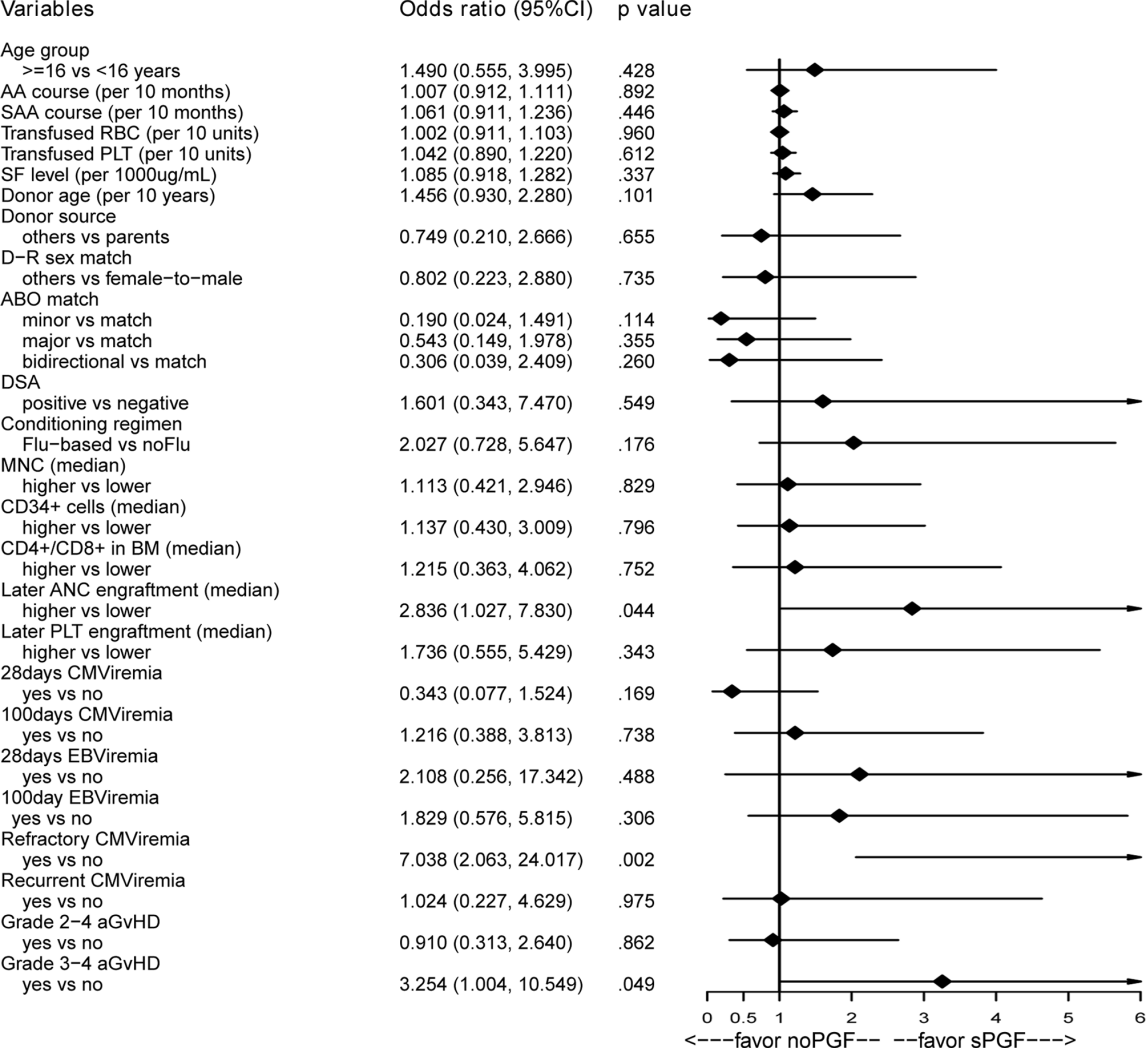
Univariable analysis of risk factors associated with sPGF.

As presented in **Figure 4**, no potential risk factor was found among the pre-transplantation variables of interest. Among the post-transplantation variables, we found that later neutrophil engraftment (OR 2.836, 95% CI [1.027, 7.830], p=.044), a history of refractory CMV viremia (OR 7.038, 95% CI [2.063, 24.017], p=.002), and a history of grade 3-4 aGvHD (OR 3.254, 95% CI [1.004, 10.549], p=.049) were associated with sPGF in the univariable analysis. No correlation was found among these variables (data not shown). When included in the multivariable analysis (**Table 2**: model 1), later neutrophil engraftment and a history of refractory CMV viremia were independent risk factors for sPGF.

**Table 2:** Multivariable analysis of factors associated with sPGF.

	p-value	Odds Ratio	95% confidence interval
Model 1^*^			
Later ANC engraftment (≥ median vs < median)	**.038**	3.027	1.063-8.622
History of refractory CMViremia (yes vs no)	**.006**	6.020	1.664-21.785
History of grade III-IV aGvHD (yes vs no)	.073		
**Model 2^†^**			
Conditioning regimen (Flu-based vs noFlu)	.208		
CD34+ cell doses (≥ median vs < median)	.314		
Later ANC engraftment (≥ median vs < median)	**.049**	2.819	1.005-7.909
History of refractory CMViremia (yes vs no)	**.002**	6.986	2.002-24.379
History of grade III-IV aGvHD (yes vs no)	.063		

sPGF secondary poor graft function, Flu fludarabine, CMViremia cytomegalovirus viremia, aGvHD acute graft versus disease. P-value <.05 was emphasized with bold fonts.

^*^The Enter method was used.

^†^The forward: LR method was used.

#### 3.4.2 Combined Analysis of Pre- and Post-Transplantation Variables

In the multivariable logistic regression model (**Table 2**: model 2), later neutrophil engraftment (OR 2.819, 95% CI [1.005, 7.909], p=.049) and refractory CMV viremia were independent risk factors for sPGF (OR 6.986, 95% CI [2.002, 24.379], p=.002). There was weak evidence that a history of grade 3-4 aGvHD was associated with sPGF (p=.063).

## 4. Discussion

PGF is a type of BM failure syndrome and leads to high morbidity and mortality post-transplantation. Based on the largest-scale AA cases that received haplo-HSCT, we revealed a CIR of 4.62% of sPGF at 3 years post-transplantation. OS was significantly decreased in sPGF patients. Later neutrophil engraftment and a history of refractory CMV viremia were the independent risk factors for sPGF.

Several studies on hematological malignancies suggested that haplo-HSCT can be associated with a greater risk of sPGF (9, 22). However, a previous report from our center demonstrated no association between haplo-HSCT setting and sPGF (23). In line with Liu et al. (24), our results reported an acceptable incidence of sPGF after haplo-HSCT for AA. In contrast, Japanese colleagues reported a higher incidence of sPGF of 15% in 49 pediatric AA patients, which included 3 transplantations from matched sibling donors (20.0%), 3 from unrelated donors (10.3%), and 1 from haploidentical donor (20%) (25). Similar to the study of Kako et al (8), Flu was suggested to be responsible for the increased incidence of sPGF, which was denied by our studies (11). The discrepancy in results can be explained by the difference in conditioning regimen instead of HSCT type, as *Liu* and we additionally applied 2 days of Bu to ensure successful engraftment and stable full donor chimerism in the haplo-setting (26). Of note, in line with prior reports, patients with high titers of DSA were successfully handled with rituximab desensitization (15, 27) and co-infusion with DSA-free cord blood (28), and as a result, none of the patient experienced primary PGF. Employing intensive conditioning regimens may be beneficial in maintaining stable donor-type chimerism and excellent hematopoietic recovery in AA patients undergoing HSCT. Further research should be conducted to clarify this hypothesis.

Widely proposed is the “seed, soil and climate” model for the pathophysiology of PGF (6, 29). Current literature suggests hematopoietic stem cells (seed) abnormalities have a causative role in PGF. In the present study, patients with sPGF or without PGF received grafts of similar dose and composition. Impressively, the dose of infused CD34+ cells was not associated with sPGF. However, we did observe distinct features of engraftment between the two groups. The univariable and multivariable analyses identified later neutrophil engraftment as an indicator of sPGF. Our results indicate that the development of sPGF is more likely the result of qualitative, rather than quantitative, abnormality of hematopoietic stem cells. Several studies demonstrated that no deficit was found in the cells’ capacity to repopulate the marrow when stored CD34+ cells from donors whose recipients developed PGF were xenografted to mice (30). Moreover, donor-derived CD34+ cells boost is an emerging therapeutic option with promising response rates in patients with sPGF, as presented in this report and others (31–33). Taken together with these findings, these data suggests that the deficits in “seed” are acquired after transplantation. Inducers or enhancers of allo-immunity (climate), such as CMV reactivation and GvHD, may amplify the intrinsic dysfunction of hematopoietic stem cells, ultimately leading to sPGF.

CMV inhibits hematopoiesis directly by infecting bone marrow or suppress hematopoiesis indirectly through the infection of stromal cells (34, 35). Previous studies (7, 23, 36) have identified CMV viremia as an independent risk factor for sPGF. It is reported that recipients undergoing haplo-HSCT have a higher incidence of CMV reactivation, as well as refractory CMV viremia (37, 38). Lv et al. recently revealed that CMV reactivation was the only hazard element for sPGF in haplo-HSCT (9). Nevertheless, one should note that refractory CMV viremia rather than early CMV reactivation increased the risk of sPGF in our study. Patients in the sPGF group had lower B cell levels in the first month after haplo-HSCT, which may explain their greater susceptibility to refractory CMV viremia (38). Our results indicated that the influence of CMV on the BM niche can be time-dependent and may be irreversible under sufficient viral load. Since the clinical course of CMV reactivation is often complicated with GvHD, administration of immunosuppressants and immune reconstitution, further study on the impact of clinical characteristics and kinetics of CMV on graft hematopoietic function is required. On the other hand, antiviral medications, including GCV and foscarnet, can exacerbate the suppression in hematopoiesis (22) and are major players in the development of sPGF. Given the fact that GCV was involved in all CMV-positive patients in this study, it was impossible to separate the influence of CMV itself and anti-CMV pharmacotherapy. Anyway, timely evaluation and initiation of CMV-specific cellular therapy may be of great help to avoid inhibition of hematopoiesis and provide better transplant outcomes (13, 39).

The occurrence of aGvHD has also been accepted as contributing to the development of sPGF (36, 40). In this study, we found a marginal association between grade 3-4 aGvHD and sPGF. *In vivo* studies corroborated that GvHD can lead to PGF *via* overactivated T cells and dysregulated cytokines (41, 42). Moreover, severe aGvHD requires intensive immunosuppression and thus always occurs in concert with prolonged viral reactivation. Limited by the number of cases, we were unable to analyze the effect of aGvHD and co-current CMV viremia on sPGF. Large-scale studies can help understand this process. Reducing the incidence of aGvHD is now one of the most important goals of unmanipulated haplo-HSCT for AA patients, but it is noteworthy that we need to develop better strategies to balance the risk of immunosuppression and viral reactivation. In the ATG-based modality of *in vivo* T cell depletion, the dose of ATG is positively related to delayed immune reconstitution and the risk of viral infection (37, 43, 44). Recent works provide a promising option by optimizing ATG dosing (2.5 mg/kg daily for 3 days) to reduce viral activation while maintaining sufficient GvHD prophylaxis in haplo-HSCT (45, 46). Similar phenomena were observed in haplo-HSCT using a combination of post-transplantation cyclophosphamide and low-dose ATG (47, 48). A lower dose of ATG (2.5 mg/kg) successfully reduces CMV reactivation without compromising the favorable effect of preventing GvHD (49). Studies should be conducted in larger cohorts to determine the optimal dose of ATG to reach maximum immunosuppression, minimum risk of severe infections and in the end the best survival.

In conclusion, sPGF can develop in 4.62% of AA patients after haplo-HSCT and significantly decreases survival. The independent hazard elements for sPGF were later neutrophil engraftment and a history of refractory CMV reactivation. Considering the limited number of sPGF cases in this report, our results warrant investigation in further studies.

## Data Availability Statement

The raw data supporting the conclusions of this article will be made available by the authors, without undue reservation.

## Ethics Statement

The studies involving human participants were reviewed and approved by the Ethics Review Committee of Peking University People’s Hospital. Written informed consent to participate in this study was provided by the participants’ legal guardian/next of kin.

## Author Contributions

LX and XH designed the study. The data analysis and manuscript development were led by FL and TH. All authors contributed to providing clinical data and approved the final version for submission.

## Funding

This work was supported by the National Key Research and Development Program of China (No. 2017YFA0104500), Innovative Research Groups of the National Natural Science Foundation of China (No. 81621001), Key Program of the National Natural Science Foundation of China (No. 81530046), and National Natural Science Foundation of China (No. 82100227).

## Conflict of Interest

The authors declare that the research was conducted in the absence of any commercial or financial relationships that could be construed as a potential conflict of interest.

## Publisher’s Note

All claims expressed in this article are solely those of the authors and do not necessarily represent those of their affiliated organizations, or those of the publisher, the editors and the reviewers. Any product that may be evaluated in this article, or claim that may be made by its manufacturer, is not guaranteed or endorsed by the publisher.
